# Ocular-cerebral immune dialogue: a new perspective and therapeutic potential of regional lymphatic systems

**DOI:** 10.3389/fimmu.2025.1595275

**Published:** 2025-08-13

**Authors:** Yiran Wang, Pei Guo, Weihong Li, Tong Li

**Affiliations:** ^1^ Basic Medical College, Chengdu University of Traditional Chinese Medicine, Chengdu, Sichuan, China; ^2^ Sichuan College of Traditional Chinese Medicine, Mianyang, Sichuan, China; ^3^ Department of Orthopedics, Hospital of Chengdu University of Traditional Chinese Medicine, Chengdu, Sichuan, China

**Keywords:** immune privilege, blood-brain barrier, central nervous system, regional lymphatic system, glymphatic system

## Abstract

The Central Nervous System (CNS), due to its unique structure and function, possesses immune privilege, which is primarily maintained through mechanisms such as the blood-brain barrier, immune cell exclusion, and neuroglial cell regulation, effectively protecting the CNS from external insults. In recent years, research has discovered the presence of functional lymphatic systems in the meninges and the posterior segment of the eye, capable of draining cerebrospinal fluid and ocular antigens to the deep cervical lymph nodes, directly connecting with the systemic immune system. This finding has revised the traditional view that the CNS lacks lymphatic circulation and has provided a new perspective for understanding CNS immune privilege. Particularly, the posterior segment of the eye shares lymphatic drainage pathways with the brain, further revealing the complex immunological connections between the two. The ocular-cerebral connected regional lymphatic system plays a key role in ocular immune surveillance and pathological links within the CNS, with its dysfunction potentially exacerbating inflammatory responses and disease progression. Moreover, this system offers new avenues for early diagnosis, immune modulation, and drug delivery in CNS diseases, demonstrating significant clinical application potential and providing a scientific basis for the diagnosis and treatment of neurodegenerative and ophthalmic diseases.

## 1 Introduction

The Central Nervous System (CNS), encompassing the brain, spinal cord, and retina, maintains immune privilege through anatomical barriers and immunoregulatory mechanisms (1–3). This concept originated in the early 20th century when studies revealed that grafts within the CNS survived longer compared to other parts of the body (4). This privilege relies on three pillars (1): BBB restricting passive immune cell entry, (2) active exclusion of pro-inflammatory lymphocytes by CNS-resident cells, and (3) neuroglial modulation of local inflammation (5). While these mechanisms protect neural homeostasis, they also impede pathogen clearance, amplifying neuroinflammatory cascades (6, 7).

For decades, the CNS was presumed to lack conventional lymphatic drainage. This dogma was overturned by 2024 breakthroughs from the Kipnis and Alitalo labs, which identified functional meningeal lymphatic vessels (mLVs) capable of shunting brain-derived antigens (e.g., α-synuclein) and immune cells to deep cervical lymph nodes (dcLNs) via cerebrospinal fluid (8). These mLVs not only establish a direct CNS-systemic immune interface but also exhibit anatomical convergence with ocular lymphatic pathways (1), hinting at an integrated ocular-cerebral immune network.

**Figure 1:**
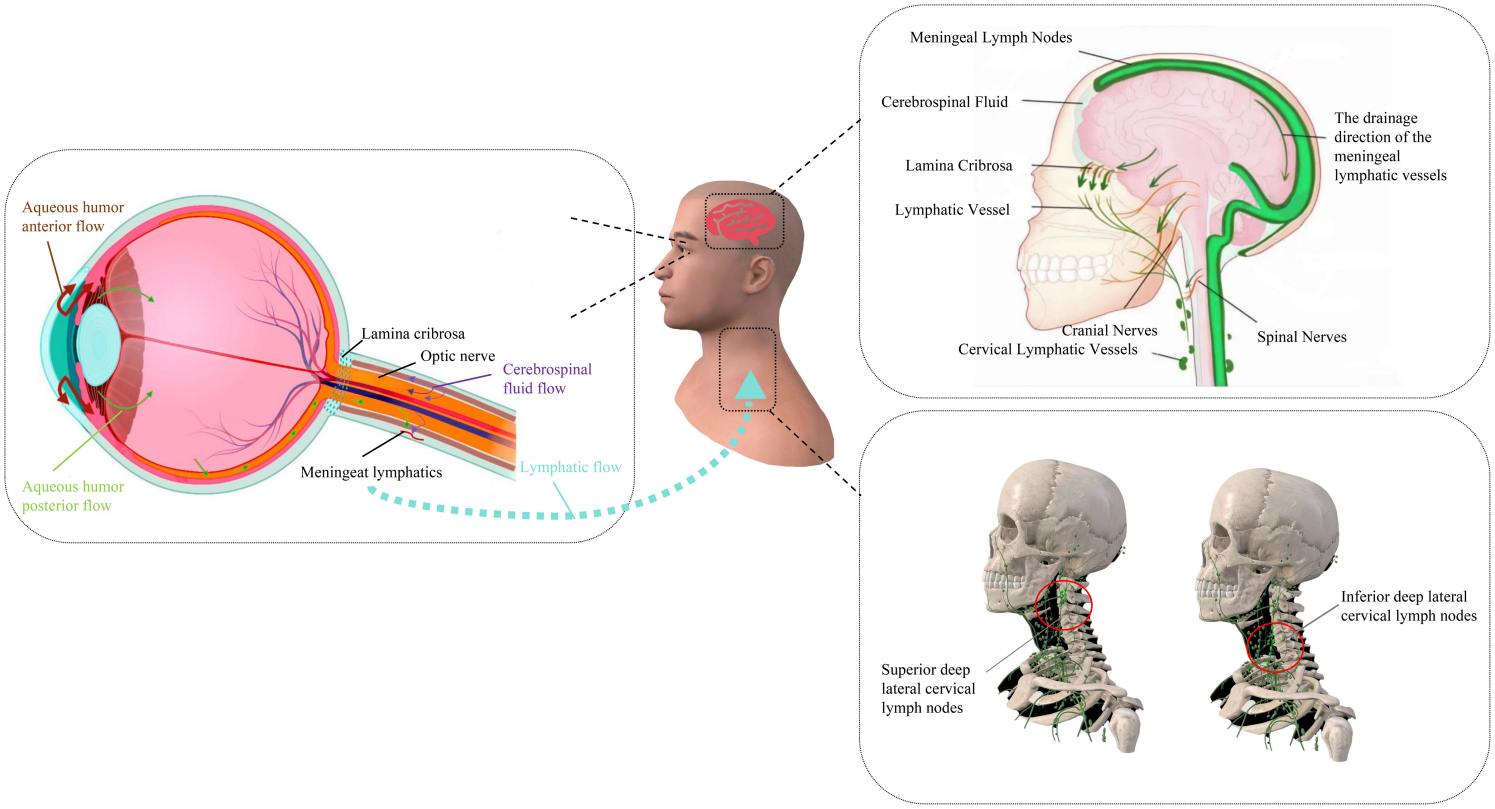
Immune links between the eye and the brain and their shared lymphatic system.

As an immune-privileged extension of the CNS, the eye not only shares lymphatic pathways with the brain but also engages in a dynamic immunological crosstalk, termed the ‘ocular-cerebral immune dialogue’ (9, 10). The retina possesses immune privilege, restricting immune cell infiltration and inflammatory responses, a characteristic crucial for protecting the retina from autoimmune diseases (11). Recent studies have, for the first time, discovered that the eye has a regional lymphatic system, especially in the posterior segment, where antigens can be drained through the lymphatic vessels of the optic nerve sheath to the deep cervical lymph nodes, which are also key drainage sites for meningeal lymphatic circulation (8). This indicates that the eye shares a lymphatic circulation system with the brain and has a close immunological connection. This finding not only expands our understanding of the immune environment of the eye but also reveals its potential role in diseases, providing new strategies for the treatment of diseases such as glaucoma and macular edema (12). (**Figure 1**) However, the molecular mechanisms underlying this shared lymphatic system and its role in bridging ocular and cerebral pathologies remain poorly understood.

In this work, we examine the emerging dual role of the ocular-cerebral lymphatic axis: as a potential pathway for neuroinflammatory pathology and a possible route for therapeutic intervention. Preliminary evidence suggests this duality: Alzheimer’s disease patients have shown concurrent tau oligomer deposition in both the retina and meningeal lymphatics (13), while preclinical studies suggest enhanced brain bioavailability of therapeutics when delivered via ocular lymphatic routes compared to systemic administration (14). Through investigating this axis, we seek to develop a conceptual framework that could support future approaches to early diagnosis (such as retinal immune biomarker profiling) and targeted therapy (including lymphatic-focused drug delivery strategies).

## 2 Literature search methodology

A systematic literature search was conducted in the Web of Science Core Collection (WoSCC) and PubMed databases on March 1, 2025, covering publications from January 2020 to March 2025. The search was limited to peer-reviewed articles, reviews, and clinical trials published in English. The search strategy employed the following query in WOSCC: TS=(((Eye Diseases OR Ophthalmopathy) AND (Meningeal lymphatics)) OR ((Central Nervous System Diseases) AND (Ocular lymphatic system))). Equivalent search terms were used in PubMed.

**Figure 2:**
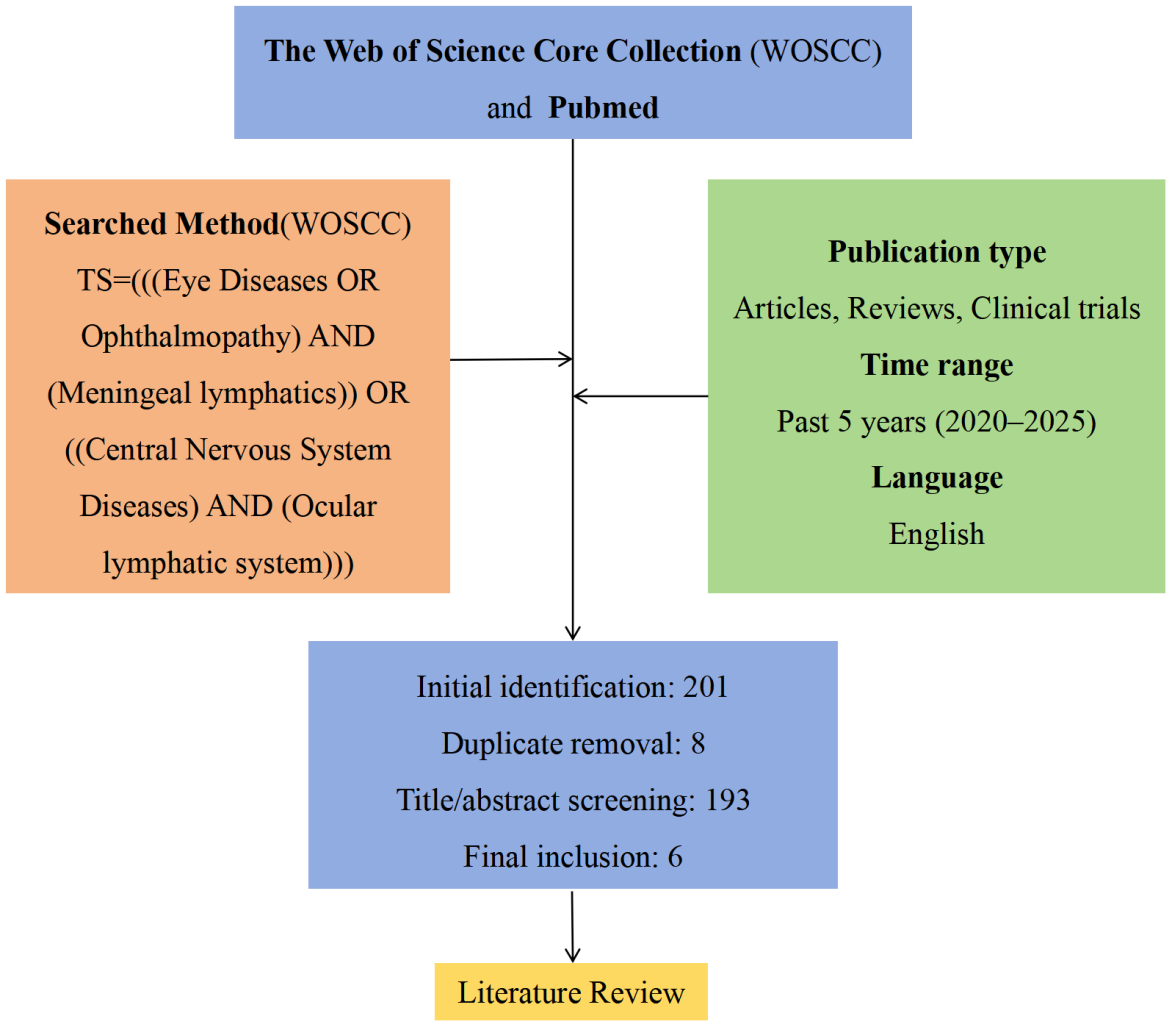
Literature screening workflow.

Two investigators independently performed the search and screened records according to predefined inclusion criteria: 1) Studies investigating ocular-cerebral lymphatic connections. 2) Research involving both ocular and central nervous system pathologies. 3) Publications containing original experimental data or comprehensive reviews. The screening process involved: Initial title/abstract screening, full-text assessment of potentially eligible studies, Final selection based on methodological quality, Any discrepancies between reviewers were resolved through discussion. The complete selection workflow is illustrated in **Figure 2** (custom PRISMA-style flowchart).

## 3 Immune privilege in the CNS and eye

Immune privilege, characterized by a restricted immune response in specific tissues or organs including the eye, brain, testes, and embryos (15–18), is crucial for protecting these vulnerable sites from damage due to their unique physiological and immunological attributes. This phenomenon allows for the fine-tuning of immune activity through barrier structures, immunosuppressive factors, and the modulation of antigen-presenting cell functions. Despite this privilege, these organs retain the capacity to recognize and eliminate pathogens, reflecting the immune system’s adaptability. Immune privilege is particularly critical for the CNS and eye, enabling them to tolerate foreign antigens without inciting excessive inflammation, which is essential for their functional preservation. Considering the limited regenerative potential of these organs, immune-mediated inflammation can result in irreversible tissue damage, further impairing their functions (19). Thus, immune privilege is vital for maintaining the stability and health of these organs (20).

The mechanisms by which the CNS and eye maintain immune privilege involve multiple aspects. Firstly, the blood-brain barrier (BBB) and the blood-retinal barrier (BRB) are two key anatomical structures that limit the entry of large molecules, immune cells, and inflammatory factors from the bloodstream into the CNS and retina, reducing the activation of immune responses and the risk of autoimmune attacks (21, 22). These barriers serve not only as physical protective measures but also play significant roles in immune modulation, helping to prevent harmful immune responses from entering these sensitive areas (23). Additionally, the microenvironment of the CNS and eye tends to repel immune cells (24). Astrocytes and Müller cells secrete anti-inflammatory factors and immunosuppressive molecules, such as Transforming Growth Factor-beta (TGF-β) and Interleukin-10 (IL-10), inhibiting the activation and proliferation of immune cells, thus playing a crucial role in maintaining immune balance and preventing excessive immune responses (25, 26). These cells secrete immunomodulatory factors to ensure that immune responses do not cause damage to neurons or retinal cells. Glial cells, particularly microglia, play a vital role in immune surveillance and modulation in the CNS (27). They not only clear pathogens and dead cells, maintaining cleanliness of the local environment, but also protect neurons from potential damage by regulating immune responses. As the primary immune cells in the CNS, microglia continuously monitor the immune status of the nervous system, promptly recognizing and responding to pathological immune responses, thereby preventing neurodegenerative damage (28). Furthermore, the CNS and eye maintain immune privilege by inducing immune tolerance, with regulatory T cells (Tregs) playing a central role. Tregs secrete anti-inflammatory cytokines, such as TGF-β and IL-10, to suppress excessive immune responses, thus avoiding attacks by the immune system on self-antigens and ensuring the maintenance of immune balance (29, 30). These mechanisms work together to effectively ensure the immunological stability of the nervous system and prevent immune damage.

In neurodegenerative diseases, immune privilege plays a crucial role. The maintenance of immune privilege not only helps to limit the occurrence of pathological inflammation but also protects neurons from damage by modulating immune responses (31). For instance, in Alzheimer’s disease (AD) and Parkinson’s disease (PD), immune privilege protects the BBB and BRB, effectively inhibiting the entry of immune cells and inflammatory factors into the CNS, thereby controlling inflammatory responses at a low level and preventing excessive damage to neurons (32). In AD, inflammatory responses are often caused by the overactivation of microglia, and maintaining the integrity of the BBB can mitigate this response and avoid destruction of neural cells (33). In PD, microglia and astrocytes protect dopaminergic neurons from damage by clearing toxic α-synuclein aggregates (34).

However, when the mechanisms of immune privilege are disrupted, the progression of neurodegenerative diseases often accelerates. Damaged or dysfunctional BBB and BRB may allow the penetration of immune cells and inflammatory factors, triggering excessive immune responses and further exacerbating neuroinflammation and neuronal damage (35, 36). The failure of immune barriers provides conditions for disease worsening, accelerating the progression of neurodegenerative diseases (31, 37). Therefore, protecting the integrity of immune privilege is an important strategy for alleviating the course of neurodegenerative diseases.

## 4 Discovery of brain lymphatic circulation: redefining CNS immunology

The discovery of brain lymphatic circulation represents a revolutionary advancement in the field of neuroscience in recent years, completely overturning the traditional perception of the CNS as “immune-privileged”. Prior to 2015, it was widely believed that the brain lacked a conventional lymphatic system, a view based on the absence of lymphatic vessels in early anatomical studies. However, research published in Nature by Jonathan Kipnis’ team in 2015 first confirmed the presence of functional lymphatic vessels within the dura mater. These lymphatic vessels are capable of draining metabolic waste and large molecular substances from cerebrospinal fluid to the deep cervical lymph nodes, providing strong evidence for a direct connection between the CNS and the systemic immune system (38–40). This finding shattered the assumption that the CNS was entirely isolated from the immune system, opening up new avenues for interdisciplinary research between neuroscience and immunology.

This discovery has redefined the connection between the CNS and the immune system. The existence of meningeal lymphatic vessels indicates that the brain is closely linked to the systemic immune system through this system. Their unique anatomical location, particularly around the dural sinuses, allows them to encounter and process antigens within the CNS before cerebrospinal fluid enters the systemic lymphatic circulation. A large number of immune cells, including antigen-presenting cells, gather near the dural sinuses, which can capture antigens from the cerebrospinal fluid and activate T cells, triggering a systemic immune surveillance mechanism (41). This mechanism maintains immunological balance in the CNS under healthy conditions and provides a new perspective for understanding immune function abnormalities under disease conditions.

Emerging research is reshaping therapeutic approaches for CNS disorders. While the blood-brain barrier has long been recognized as a major obstacle for CNS drug delivery, recent studies suggest that meningeal lymphatic vessels may represent an alternative potential pathway. Preliminary evidence indicates that modulation of these lymphatic vessels could potentially enhance CNS delivery of certain therapeutics, particularly for compounds that demonstrate poor blood-brain barrier penetration. However, the clinical applicability of this approach requires further validation (42–44). In addition, the immune surveillance function of meningeal lymphatic vessels provides possibilities for early diagnosis and intervention of diseases. For instance, by monitoring immune changes in the meningeal lymphatic vessels, it may be possible to capture early signals of diseases, enabling more timely treatment (45–47). Furthermore, modulating the function of meningeal lymphatic vessels also offers a new direction for the treatment of CNS diseases. In diseases such as Alzheimer’s disease (AD) and Parkinson’s disease (PD), the drainage function of meningeal lymphatic vessels can be used to clear the accumulation of proteins like amyloid-beta (Aβ) or α-synuclein, thereby reducing inflammatory responses (48–50). This finding provides new ideas for immunotherapy strategies, achieving precise intervention in diseases by modulating specific immune pathways.

The discovery of the glymphatic system has further enriched our understanding of CNS metabolic clearance mechanisms. The glymphatic system, a brain-wide paravascular pathway facilitated by astrocytic endfeet and the water channel aquaporin-4 (AQP4), is responsible for the clearance of interstitial solutes and metabolic waste from the brain parenchyma into the cerebrospinal fluid (CSF). This system operates in concert with the meningeal lymphatic vessels, which drain CSF and its contents into the deep cervical lymph nodes (dcLNs). The synergistic action of the glymphatic system and meningeal lymphatic vessels not only ensures efficient clearance of CNS waste but also maintains the sensitivity of immune surveillance while avoiding interference with the microenvironment of neurons (51, 52). This complex clearance and immune regulation system provides new insights into understanding brain health and disease and may offer key breakthroughs for the treatment of various neurological diseases.

Therefore, the discovery of brain lymphatic circulation has deepened our understanding of CNS immunology and redefined the connection between the CNS and systemic immunity. This advancement provides a new perspective for the early diagnosis and treatment of CNS diseases, while revealing potential therapeutic targets and strategies.

## 5 Ocular lymphatic system

The ocular lymphatic system, a significant discovery in the field of immunology in recent years, has revealed the critical role of lymphatic drainage systems in different regions of the eye in immune responses (8). This system is not only of great importance for the diagnosis and treatment of ocular diseases but also provides a new perspective for understanding the interaction between the eye and the systemic immune system. Through this discovery, we can gain a deeper understanding of the complex relationship between local ocular immune functions and systemic immune regulation.

The lymphatic drainage system of the eye can be divided into two main regions: the anterior and the posterior. The anterior region includes the cornea, iris, and ciliary body, while the posterior region primarily refers to the retina and choroid (53). These two regions have significant structural and functional differences and undertake different immune regulatory functions. The lymphatic drainage of the anterior segment of the eye mainly relies on the aqueous humor and the lymphatic vessels of the anterior chamber angle. Aqueous humor, the transparent fluid filling the anterior chamber, is not only responsible for the nutritional supply of the cornea and lens but also participates in the clearance of metabolic waste. Aqueous humor enters the Schlemm’s canal through the trabecular meshwork at the anterior chamber angle and eventually drains into the peripheral venous system (54). In this process, not only are metabolic wastes cleared, but local immune responses are also regulated. The lymphatic system of the anterior segment plays an important role in the occurrence of glaucoma and other intraocular pressure-related diseases, increased intraocular pressure may lead to the obstruction of aqueous humor outflow, thereby affecting the balance of the local immune environment (55). Compared to the anterior segment, the lymphatic system of the posterior segment of the eye is more complex. The lymphatic drainage of the retina and choroid mainly occurs through the lymphatic vessels of the optic nerve sheath, these lymphatic vessels drain antigens and waste from the eye to the deep cervical lymph nodes and are directly connected to the systemic immune system (56). This mechanism indicates that the lymphatic system of the posterior segment of the eye may play a crucial role in regulating ocular immune responses, especially in immune responses related to retinal diseases such as age-related macular degeneration and retinal vascular diseases.

The ocular lymphatic system has a multifaceted impact on immune responses in ocular diseases. Firstly, the posterior lymphatic system participates in systemic immune surveillance by draining ocular antigens to the deep cervical lymph nodes, maintaining ocular immune balance. This mechanism is of great importance in preventing autoimmune diseases (such as uveitis), and lymphatic system dysfunction may lead to immune surveillance disorders, thereby triggering or exacerbating ocular inflammation (8). Secondly, the anterior lymphatic system also plays a significant role in pathogen clearance, especially in infectious keratitis, where the circulation and drainage of aqueous humor are crucial for clearing pathogens and inflammatory factors. If this process is obstructed, it may lead to the spread of infection and exacerbation of inflammation (57, 58). Additionally, the occurrence of ocular diseases such as dry eye syndrome may be closely related to lymphatic system dysfunction. In patients with dry eye syndrome, there is a link between the inflammatory response of the lacrimal glands and the abnormal regulation of the lymphatic system, such abnormal regulation may lead to a decrease in tear secretion, thereby causing ocular surface inflammation and affecting ocular health (9). Studies have also found that the lymphatic system of the posterior segment of the eye is closely related to the occurrence and progression of neurodegenerative eye diseases. In age-related macular degeneration (AMD), the immune response of the retina may be closely related to lymphatic system dysfunction, and the lymphatic system may be involved in the clearance of retinal pigment epithelial cells and the regulation of inflammatory factors, thereby affecting the progression of the disease (59). Therefore, the ocular lymphatic system plays an important and complex role in ocular immune responses. A deep understanding of the functions and regulatory mechanisms of this system is of great significance for the development of new treatments for ocular diseases, especially those related to immune dysfunction.

## 6 Immunological connection between the eye and brain

The immunological connection between the eye and the brain is an intricate biological process and an emerging research focus in the field. It reveals the complex interplay between the two organ systems, primarily mediated by the ocular-cerebral connected regional lymphatic system. This article highlights the regional lymphatic system that drains antigens and waste from the eye to the deep cervical lymph nodes through the optic nerve sheath lymphatics. Notably, these lymph nodes are also key drainage sites for the meningeal lymphatic circulation. This direct anatomical link suggests that immunological changes in the eye can rapidly reflect in the brain, and the immunological status of the brain may also affect the eye. This unique immunological connection provides a new perspective for studying the interaction between the eye and brain, especially in the context of neurodegenerative diseases, where its potential role is more significant.

In diseases such as Alzheimer’s disease (AD) and multiple sclerosis (MS), there is a significant similarity in pathological features between the brain and the eye. Pathological changes in the retina of AD patients are highly consistent with those in the brain (60, 61), suggesting that the retina may serve as an important biomarker reflecting the brain’s disease state. Monitoring immunological changes in the retina could facilitate early diagnosis and intervention for neurodegenerative diseases. Similarly, demyelinating pathological changes in the retina of MS patients have been observed (62, 63), further confirming the immunopathological link between the eye and the brain. These findings lay the foundation for using the eye as a window to study central nervous system diseases.

Moreover, the ocular lymphatic system is not only a hub for immune surveillance but may also influence the progression of neurodegenerative diseases. In neurodegenerative diseases, inflammatory responses are often a key factor, they may be triggers for the disease or accelerate disease progression (64–67). By modulating the function of the ocular lymphatic system, it may be possible to reduce the entry of immune cells and inflammatory factors into the brain, thereby alleviating local inflammatory responses and slowing disease progression (68). The discovery of this mechanism provides a new direction for the treatment of neurodegenerative diseases. Traditionally, the blood-brain barrier is considered the main obstacle for CNS drug therapy (69). However, the ocular lymphatic system may serve as a new pathway for delivering therapeutic agents to the brain. This pathway can bypass the blood-brain barrier, directly targeting the brain and thus enhancing the specificity and efficacy of treatment, which is particularly significant for diseases that are difficult to penetrate the blood-brain barrier.

In the realm of therapeutic strategies, the immunological connection between the eye and the brain presents intriguing possibilities. It is hypothesized that by modulating the drainage function of the lymphatic system in the posterior segment of the eye, there may be a potential to enhance the clearance of pathological protein aggregates such as amyloid-beta(Aβ) in Alzheimer’s disease (AD) or α-synuclein (α-syn) in Parkinson’s disease (PD). These proteins are known to be associated with disease progression. While this hypothesis suggests that such a clearance mechanism could potentially reduce brain inflammation and protect neurons from damage, thereby possibly delaying disease progression (70), it is important to note that this is currently speculative.

The idea that targeting the lymphatic system could provide a means to develop new immunotherapeutic strategies is an area that warrants further exploration. By modulating specific immunological pathways, it may be possible to intervene in the pathological progression of diseases. However, it is crucial to emphasize that this approach is still in the realm of hypothesis and requires substantial experimental evidence from both preclinical models and clinical trials to be validated.

We acknowledge the lack of empirical validation at this stage and the need for future research to explore these possibilities. The potential of this approach to offer novel targets for immunotherapeutic strategies is an area that we believe deserves further investigation, with the understanding that it is not yet a proven treatment strategy.

## 7 Ocular-cerebral connected regional lymphatic system: therapeutic potential in disease treatment

**Figure 3:**
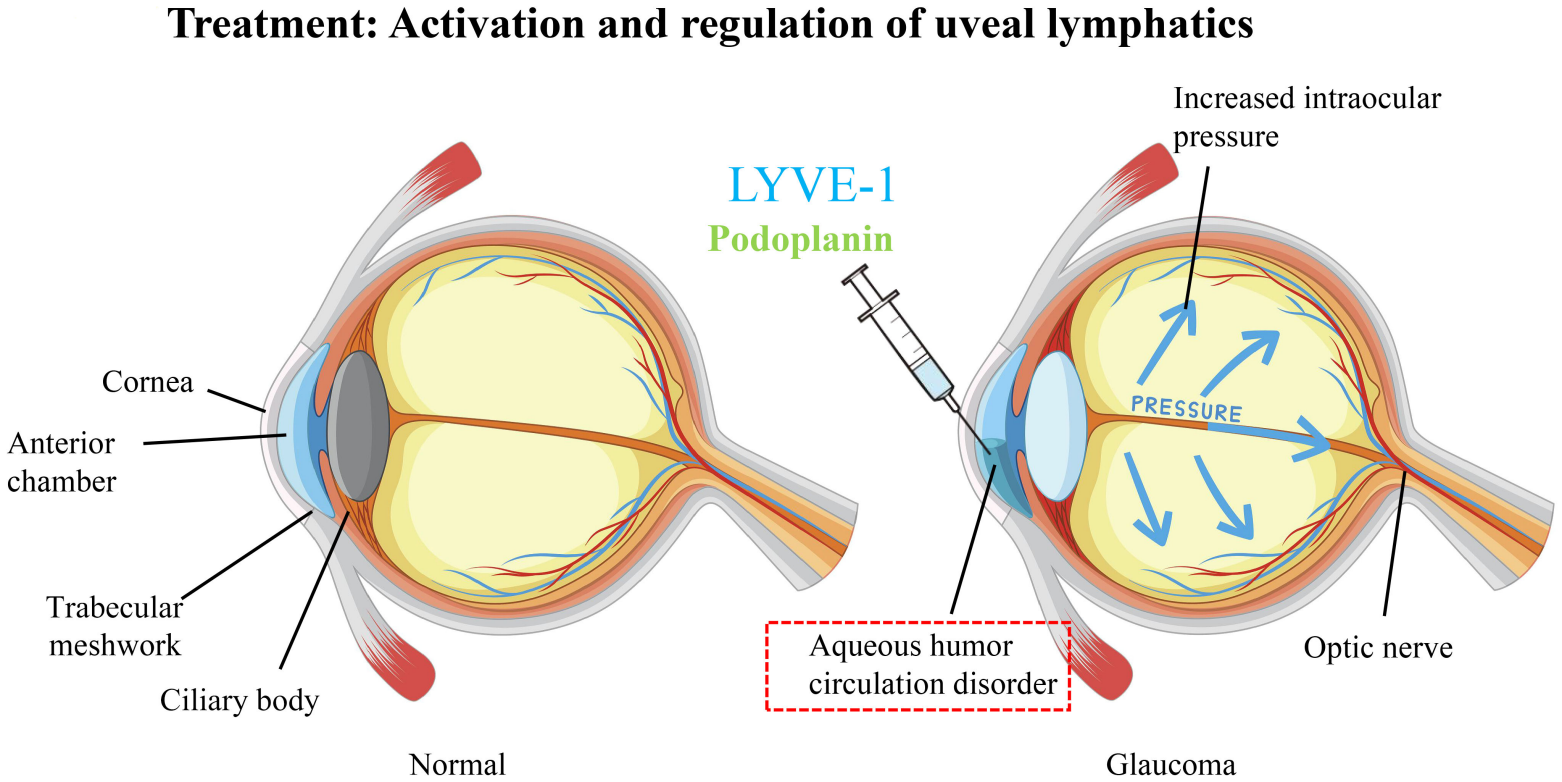
Treatment: activation and regulation of the uveal lymphatic system.

The ocular-cerebral connected regional lymphatic system holds significant therapeutic potential, particularly in the treatment of ocular diseases and central nervous system (CNS)-related conditions. This system offers innovative avenues for treating ocular diseases such as glaucoma, macular edema, and age-related macular degeneration (AMD). In glaucoma, elevated intraocular pressure is often due to the obstruction of aqueous humor outflow, affecting the balance of the local immune environment. A study has shown that using specific lymphatic markers (71) such as podoplanin and lymphatic vessel endothelial hyaluronan receptor-1 (LYVE-1) can more clearly trace the drainage pathway of aqueous humor within the eye, known as the uveal lymphatics, which are responsible for draining aqueous humor to the extraocular lymphatic system. Activating and modulating the function of uveal lymphatics may promote the effective drainage of aqueous humor, reduce intraocular pressure, and alleviate optic nerve damage. (**Figure 3**).

**Figure 4:**
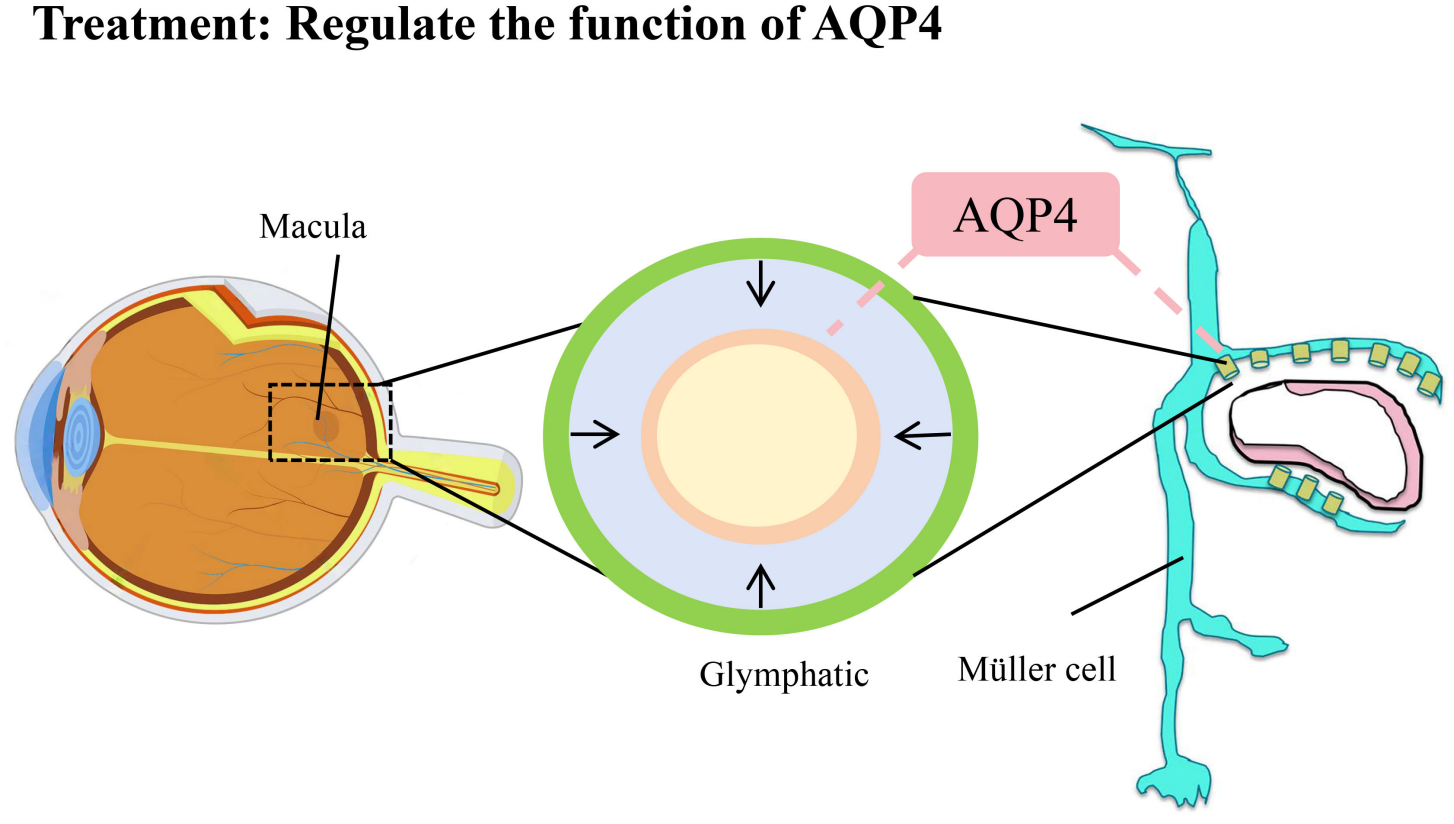
Treatment of macular edema: regulate the function of AQP4.

Additionally, the occurrence of macular edema is closely related to the function of the “glymphatic” system within the retina. This system, discovered in the brain in recent years, is a type of lymphatic system that depends on the expression of aquaporin-4 (AQP4) in Müller glial cells to clear waste and excess fluid, maintaining the fluid balance of the retina. A collaborative study between Paris Descartes University and the University of Lausanne found that when the “glymphatic” system is impaired, it may lead to the accumulation of fluid in the macular region, triggering macular edema. In terms of treatment, modulating the function of AQP4 and improving fluid clearance mechanisms may alleviate macular edema and protect vision (72). Although there is currently no direct evidence showing that the ocular lymphatic system directly improves macular edema, the concept of the “glymphatic” system provides a new perspective for research. Further exploration of the impact of this system on retinal fluid dynamics will help us understand the potential role of the ocular lymphatic system in macular edema and may reveal new therapeutic pathways. (**Figure 4**).

**Figure 5:**
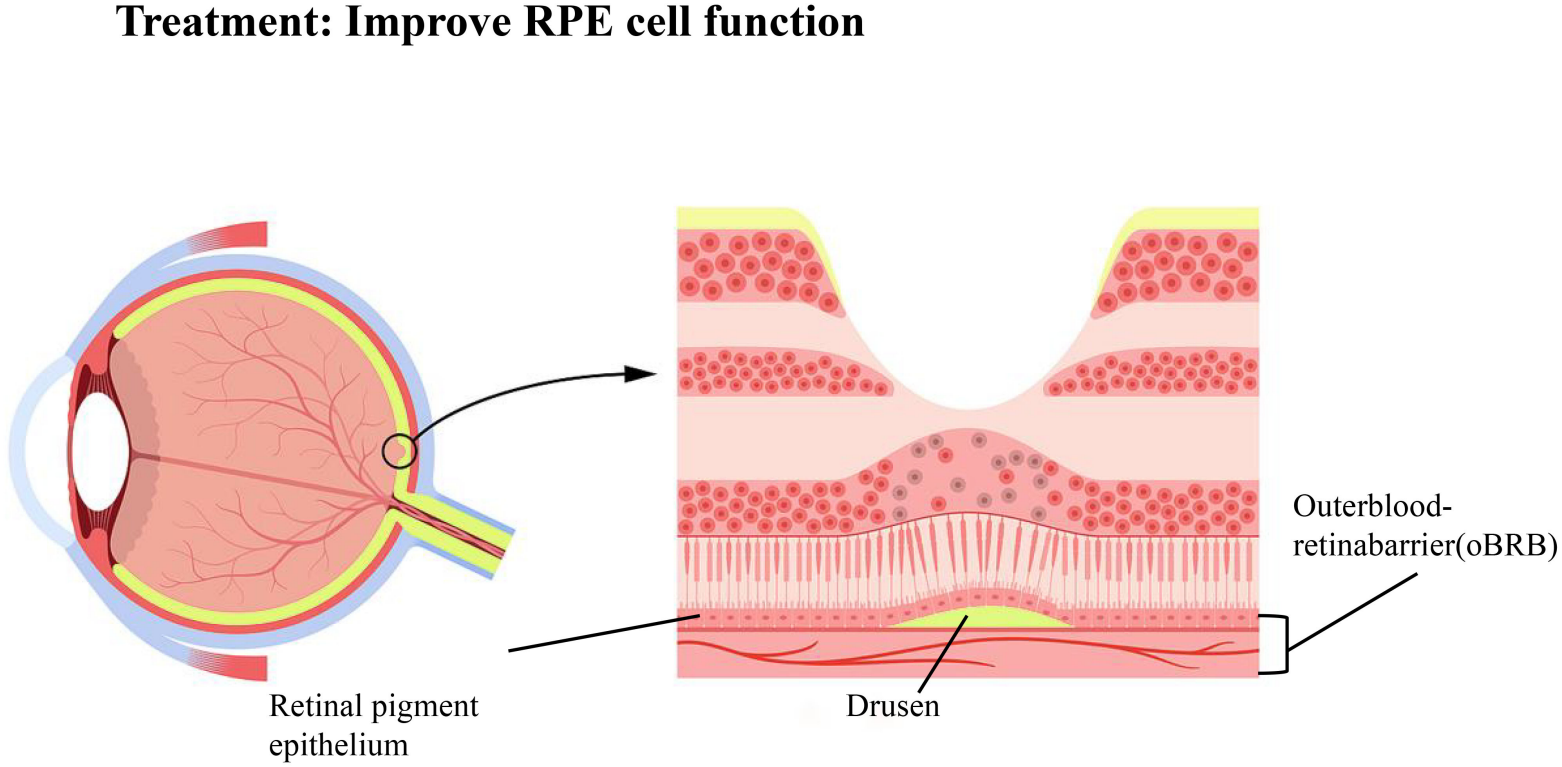
Treatment: Improve RPE cell function.

AMD is a multifactorial retinal disease involving aging, oxidative stress, genetics, environmental factors, and immune responses. Its development is associated with the obstruction of the ocular glial lymphatic system, which may affect the clearance of metabolic waste and the regulation of inflammatory factors, leading to a decline in the clearance function of retinal pigment epithelial cells (RPE) and exacerbating retinal damage (**Figure 5**). Additionally, the aging process also affects the clearance efficiency of the glial lymphatic system, which is an important risk factor for the development of AMD (73). Therefore, improving the function of the ocular glial lymphatic system is crucial for maintaining ocular health.

**Figure 6:**
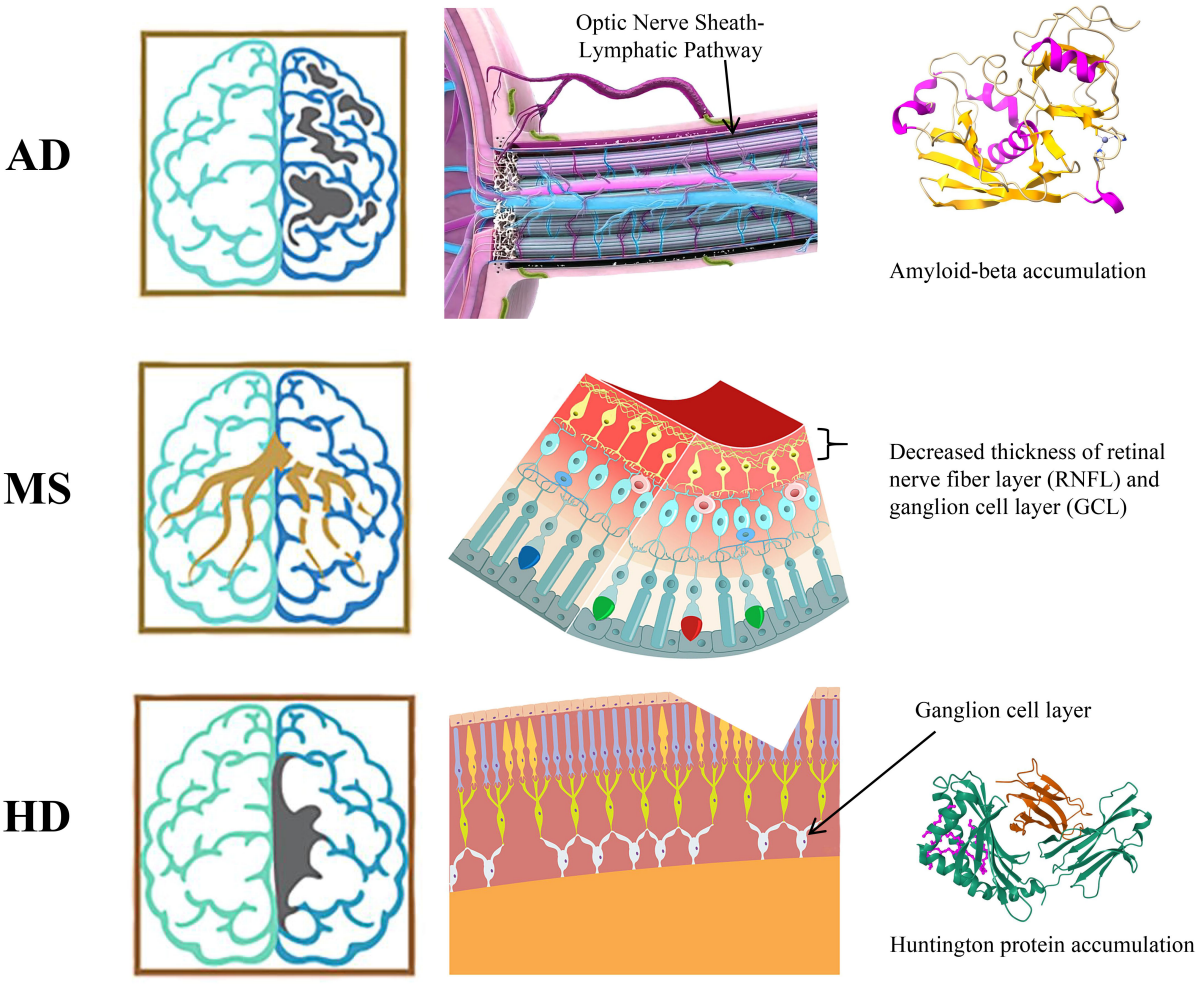
Ocular pathological changes of CNS diseases.

Furthermore, the ocular-cerebral connected regional lymphatic system is closely related to the improvement of treatment strategies for CNS diseases. In Alzheimer’s disease (AD), a team from Nanjing Medical University led by Liu Qinghuai and Xiao Ming found that amyloid-beta (Aβ) in cerebrospinal fluid can be transported to the eye through three brain-eye pathways: the optic nerve sheath lymphatic pathway, the axonal gap pathway, and the periarterial space pathway, and can affect brain-eye transport and retinal lymphatic clearance systems and AD-related ocular pathological changes by regulating AQP4 (74). This research not only explains the source, flow path, and pathological changes caused by Aβ in the eye during the progression of Alzheimer’s disease but also explores new pathogenic mechanisms and therapeutic targets for eye and brain diseases from a combined perspective of brain and eye. In patients with multiple sclerosis (MS), non-invasive optical coherence tomography (OCT) technology has shown that the thinning of the retinal nerve fiber layer (RNFL) and the ganglion cell layer (GCL) is closely related to disease progression (75). In particular, the reduction in the volume of the retinal ganglion cell-inner plexiform complex (GCIPL) is associated with an increase in the number of B cells in the meninges, becoming an independent risk factor for the worsening of disability (76). This suggests that changes in the retinal layers may reflect immune activity within the CNS. Peripheral B cells capture and present CNS antigens to activate T cells, spread CNS antigens to the periphery, disrupt the integrity of the blood-retinal barrier (BRB), and weaken immune tolerance, thereby activating autoreactive T cells and further exacerbating CNS inflammation and affecting the ocular lymphatic system. These complex interactions provide a new perspective for the diagnosis and treatment of MS, emphasizing the importance of targeted therapeutic strategies that address the interaction between B cells and T cells (77). Huntington’s disease (HD) is a neurodegenerative disease characterized by the abnormal aggregation of mutated HTT protein in the brain and neuronal damage. Studies have found that the retina of HD patients also shows abnormal changes, especially an increase in the aggregation of mutated HTT protein in the ganglion cell layer (78), similar to the aggregation of HTT protein in the brain, suggesting that the retina is a potential window for monitoring the progression of HD. The ocular glial lymphatic system plays a key role in clearing retinal HTT protein aggregates and maintaining retinal health. Research has shown that the Rho-associated kinase inhibitor HA-1077, delivered via liposomal intravenous administration, can effectively reach the retina, reduce the death of retinal photoreceptor cells in the HD transgenic mouse model R6/2, enhance the amplitude of the electroretinogram (ERG) (79), and alleviate the degeneration of retinal neurons, providing a new strategy for the treatment of HD and emphasizing the potential therapeutic value of improving the function of the ocular glial lymphatic system to clear HTT protein aggregates.(**Figure 6**).

**Figure 7:**
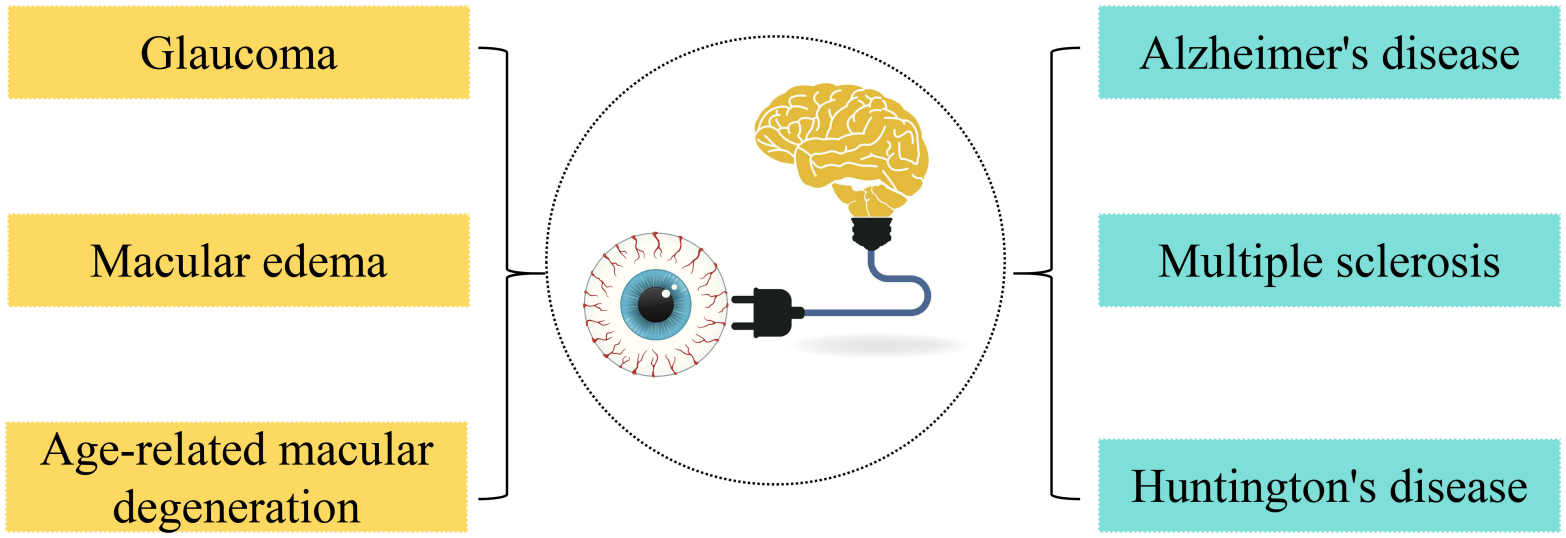
Potential of the regionally localized lymphatic system in the treatment of disease.

Therefore, emerging evidence suggests the ocular-cerebral connected regional lymphatic system may contribute to the treatment of ocular diseases and central nervous system (CNS) disorders. Current understanding indicates it serves as an important immune modulation center and may offer a potential pathway to circumvent the blood-brain barrier. This could theoretically enable more efficient delivery of therapeutic agents to the brain and support early disease detection and targeted treatment approaches, though these applications require further clinical validation.(**Figure 7**).

## 8 Synthesis and future directions

### 8.1 Ocular-cerebral immune dialogue: current consensus

This article explores the complex immunological connections between the eye and the brain, emphasizing the central role and clinical significance of the ocular-cerebral connected regional lymphatic system. With the discovery of the brain’s lymphatic circulation, the traditional notion of the CNS as “immune-privileged” has been completely overturned. This finding not only confirms that the CNS possesses a dedicated lymphatic circulation but also reveals its direct interaction with the systemic immune system. Under healthy conditions, this system maintains homeostasis in the CNS through the clearance of metabolic waste and immune surveillance; whereas in disease states, such as Alzheimer’s disease (AD) and multiple sclerosis (MS), its dysfunction may trigger inflammation and accelerate pathological progression. The discovery of the glymphatic system further deepens our understanding of the CNS’s metabolic clearance mechanisms, particularly providing a critical breakthrough in the clearance of harmful substances like amyloid-beta (Aβ), opening up new perspectives for the treatment of neurodegenerative diseases like AD.

The ocular lymphatic system, especially the posterior segment of the eye, plays a significant role in maintaining ocular immune surveillance and pathological connections with the CNS. Studies suggest that dysfunction of this system may lead to ocular diseases such as glaucoma and macular edema, and its pathological changes may also reflect the immune status of the CNS. For instance, the accumulation of Aβ in the retina or demyelinating lesions can not only indicate pathological features of the CNS but may also serve as early biomarkers for AD or MS. More importantly, by modulating the lymphatic drainage function of the posterior segment of the eye, it may be possible to promote the clearance of metabolic waste from the retina and brain, alleviate inflammatory responses, and slow the progression of neurodegenerative diseases. The clinical significance of this mechanism is profound. On one hand, immune changes in the eye provide an important window for the early diagnosis of CNS diseases; on the other hand, its unique lymphatic drainage function also offers a new pathway for drug delivery across the blood-brain barrier. Therefore, delving deeper into the specific roles and regulatory mechanisms of the ocular-cerebral connected regional lymphatic system in diseases will lay the foundation for the development of personalized medicine and treatment, promoting more precise diagnostics and intervention strategies. These findings not only provide us with new perspectives but also offer innovative methods for the early diagnosis and treatment of CNS diseases.

### 8.2 Translational challenges and interspecies variability

However, it is crucial to acknowledge the significant translational hurdles that must be addressed when considering the application of these findings from preclinical models to human clinical practice. While animal models, particularly rodents, have provided invaluable insights into the ocular-cerebral lymphatic system, there are notable anatomical, architectural, and immunological differences between rodents and humans that may limit the direct applicability of these findings. For instance, the lymphatic architecture in the human meninges and ocular tissues may differ in complexity and function compared to that in rodents. Additionally, the immune microenvironment, including the distribution and activity of immune cells, may vary significantly between species. These differences could affect the efficacy and safety of therapeutic interventions targeting the ocular-cerebral lymphatic system.

Moreover, the translation of therapeutic strategies from animal models to humans also requires careful consideration of the potential for interspecies variability in the response to treatments. For example, the efficacy of lymphatic modulation or drug delivery systems that work well in rodent models may not directly translate to humans due to differences in lymphatic drainage pathways, immune cell populations, and the overall immune response. Therefore, future research must include detailed comparative studies to elucidate these differences and develop strategies that can effectively bridge the gap between preclinical and clinical applications.

### 8.3 The eye as a window for early diagnosis

Furthermore, the concept of the eye serving as a window for the early diagnosis of CNS diseases is greatly facilitated by the shared drainage lymph nodes between the eye and the brain. This shared mechanism suggests that monitoring immunological changes in the eye can capture early signals of CNS diseases. For instance, pathological features appearing in the retina of AD patients are similar to those in the brain, making the retina a potential mirror of brain disease status. This diagnostic approach offers the possibility of timely recognition and intervention before the manifestation of disease symptoms, which could improve the success rate of early treatment and, consequently, the prognosis for patients.

### 8.4 Potential of ocular-cerebral lymphatic system in CNS disease treatment

Recent research further reveals the potential importance of the ocular-cerebral connected regional lymphatic system in the treatment of CNS diseases. In February 2024, a research team from Yale University discovered that the compartmentalized lymphatic system within the eye can mediate ocular-cerebral immune interactions. In mouse models, when the brain is infected with herpes simplex virus, the immune response within the vitreous body of the eye can protect the brain from viral invasion (8). This protective mechanism is not limited to viral infections but also applies to bacterial infections and tumors, indicating that ocular immune responses have a broad protective effect on the brain. Additionally, the role of ocular lymphatic drainage in gene therapy should not be overlooked. Studies have shown that injecting recombinant adeno-associated virus (AAV) behind the eye can more effectively elicit an immune response, which is crucial for the immune response in gene therapy (80, 81). Based on this discovery, using the ocular lymphatic system to deliver gene therapy agents to the brain may open up new avenues for the treatment of CNS diseases. In particular, blocking VEGFR-3 to suppress the immune response to AAV can reduce the risk of immune rejection in gene therapy, enabling multiple gene deliveries (82, 83), thereby improving the efficiency and effectiveness of gene therapy and providing safer and more efficient treatment plans for CNS diseases. The shared lymphatic circulation between the eye and the brain plays a key role in CNS immune responses, which is not only significant for the pathogenesis of diseases but also provides new potential targets for treatment strategies. Modulating this immune system may help control inflammatory responses, thus slowing the progression of neurodegenerative diseases. These findings provide new treatment ideas for diseases that traditional therapies struggle to cross the blood-brain barrier and lay the scientific foundation for exploring innovative treatment pathways.

### 8.5 Contradictory evidence and unresolved questions

However, some studies have presented findings that contradict the prevailing perspective on the role of meningeal lymphatics in amyloid clearance. For example, a Finnish scientific team demonstrated that genetic manipulation of dura mater lymphatics in AD mice, through either atrophy or expansion of meningeal lymphatic vessels (dLVs) using VEGF-C/VEGF-D traps or VEGF-C overexpression, affected cerebrospinal fluid (CSF) drainage but did not alter the brain’s Aβ plaque load or behavioral phenotypes (84). This finding challenges the notion that dLVs are a primary route for Aβ clearance, suggesting that other compensatory mechanisms may be at play.

The study by Antila et al. (84) revealed that Aβ deposits in the dura mater are primarily associated with bridging veins connecting the brain to large dural sinuses, rather than with dLVs themselves. This indicates that these blood vessels and/or their perivascular spaces might be more directly involved in the transport of brain-derived Aβ into the meningeal layers. Moreover, despite the impairment of dLV function, CSF drainage into the blood circulation was improved, likely through compensatory pathways such as direct efflux through the blood-CSF barrier or perivascular routes.

These findings highlight the complexity of CSF clearance mechanisms and suggest that the role of dLVs in Aβ clearance may be more limited than previously thought. Future research should explore other plausible drainage or immune-modulatory mechanisms, such as the roles of perivascular routes, astrocytic endfeet, and microglial activity in Aβ clearance, as well as the interplay between dLVs and other immune cells in the context of neuroinflammation. Of course, the discovery process of brain lymphatic circulation continues, and these new findings prompt the scientific community to re-evaluate the potential of meningeal lymphatics as therapeutic targets.

### 8.6 Future research priorities

Therefore, when discussing future research directions, these key scientific questions need to be further addressed. First, we need to delve deeper into the physiological functions and mechanisms of the ocular-cerebral connected regional lymphatic system, including detailed anatomical studies of the lymphatics, exploring how they affect the transport of immune cells and antigen presentation, and their specific roles in diseases. Second, the association of this system with CNS diseases, including its specific role in the occurrence and development of diseases, also involves the development of new biomarker detection methods and the validation of these biomarkers in predicting and monitoring disease progression. In addition, research is needed on how to translate these basic scientific findings into clinical applications, developing new therapeutic strategies and new diagnostic tools, including customizing personalized treatment plans based on the patient’s specific immune status and disease progression, achieving early diagnosis and precise intervention.

Although the ocular-cerebral connected regional lymphatic system provides us with a new perspective for understanding CNS diseases and may bring revolutionary progress in the diagnosis and treatment of these diseases, we must acknowledge that this field is still in its infancy, lacking extensive experimental evidence, case studies, and clinical data as support. This limitation means that our conclusions need to be further consolidated in future studies through more experiments and clinical validations. Therefore, we call on the scientific community to conduct more research to fill these knowledge gaps and provide a solid scientific foundation for this emerging field.

## 9 Conclusion

Current research is revealing important immunological connections between the eye and the brain, which appear to contribute to the immune privilege of both the central nervous system (CNS) and ocular tissues. These findings may offer new opportunities for early detection of neuro-ophthalmic diseases and potential therapeutic approaches. Emerging evidence suggests that monitoring and potentially modulating these connections could lead to improved interventions for neurodegenerative diseases and ocular disorders. The ocular-cerebral lymphatic system appears to play a significant role in immune regulation, with preliminary data indicating its involvement in clearing pathological protein aggregates and modulating inflammatory responses. Further investigation of this system’s functions and mechanisms could provide important insights for diagnostic and therapeutic development. This review aims to examine the potential roles of the ocular-cerebral lymphatic system in disease contexts, with the goal of informing future research toward more targeted treatment strategies for CNS and ophthalmic conditions.
